# 4-Chloro-*N*-cyclo­hexyl­benzamide

**DOI:** 10.1107/S1600536809021217

**Published:** 2009-06-10

**Authors:** Aamer Saeed, Rasheed Ahmad Khera, Muhammad Latif, Masood Parvez

**Affiliations:** a Department of Chemistry, Quaid-i-Azam University, Islamabad 45320, Pakistan; b Department of Chemistry, Hamdard Institute of Pharmaceutical Sciences, Hamdard University–Islamabad Campus, Islamabad, Pakistan; c Department of Chemistry, The University of Calgary, 2500 University Drive NW, Calgary, Alberta, Canada T2N 1N4

## Abstract

In the title compound, C_13_H_16_ClNO, the cyclo­hexyl ring adopts a chair conformation, with puckering parameters *Q* = 0.576 (3) Å, θ = 0.1 (3) and ϕ = 8 (15)°. In the crystal structure, inter­molecular N—H⋯O hydrogen bonds link mol­ecules into one-dimensional chains propagating in [010].

## Related literature

For applications of *N*-substituted benzamides, see: Beccalli *et al.* (2005 ▶); Calderone *et al.* (2006 ▶); Vega-Noverola *et al.* (1989 ▶); Zhichkin *et al.* (2007 ▶); Lindgren *et al.* (2001 ▶); Olsson *et al.* (2002 ▶). For related crystal structures, see: Jones & Kuś (2004 ▶); Saeed *et al.* (2008 ▶). For puckering parameters, see: Cremer & Pople (1975 ▶). For a description of the Cambridge Structural Database, see: Allen (2002 ▶). 
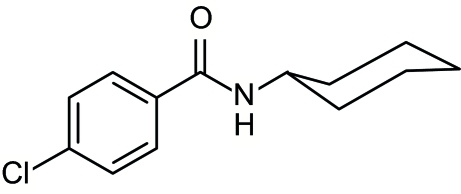


## Experimental

### 

#### Crystal data

C_13_H_16_ClNO
*M* *_r_* = 237.72
Monoclinic, 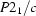

*a* = 14.755 (14) Å
*b* = 5.043 (7) Å
*c* = 16.818 (16) Å
β = 96.13 (6)°
*V* = 1244 (2) Å^3^
*Z* = 4
Mo *K*α radiation
μ = 0.29 mm^−1^
*T* = 173 K
0.12 × 0.08 × 0.06 mm

#### Data collection

Bruker APEXII CCD diffractometer
Absorption correction: multi-scan (*SORTAV*; Blessing, 1997 ▶) *T*_min_ = 0.967, *T*_max_ = 0.983
3651 measured reflections
2388 independent reflections
1497 reflections with *I* > 2σ(*I*)
*R*_int_ = 0.034

#### Refinement

*R*[*F* ^2^ > 2σ(*F* ^2^)] = 0.051
*wR*(*F* ^2^) = 0.151
*S* = 1.06
2388 reflections
145 parameters
H-atom parameters constrained
Δρ_max_ = 0.16 e Å^−3^
Δρ_min_ = −0.21 e Å^−3^

### 

Data collection: *COLLECT* (Hooft, 1998 ▶); cell refinement: *DENZO* (Otwinowski & Minor, 1997 ▶); data reduction: *SCALEPACK* (Otwinowski & Minor, 1997 ▶); program(s) used to solve structure: *SHELXS97* (Sheldrick, 2008 ▶); program(s) used to refine structure: *SHELXL97* (Sheldrick, 2008 ▶); molecular graphics: *ORTEP-3 for Windows* (Farrugia, 1997 ▶); software used to prepare material for publication: *SHELXTL* (Sheldrick, 2008 ▶).

## Supplementary Material

Crystal structure: contains datablocks global, I. DOI: 10.1107/S1600536809021217/lh2834sup1.cif

Structure factors: contains datablocks I. DOI: 10.1107/S1600536809021217/lh2834Isup2.hkl

Additional supplementary materials:  crystallographic information; 3D view; checkCIF report

## Figures and Tables

**Table 1:** Hydrogen-bond geometry (Å, °)

*D*—H⋯*A*	*D*—H	H⋯*A*	*D*⋯*A*	*D*—H⋯*A*
N1—H1⋯O1^i^	0.88	2.06	2.901 (5)	160

Symmetry code: (i) 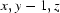.

**Fig. 1.**
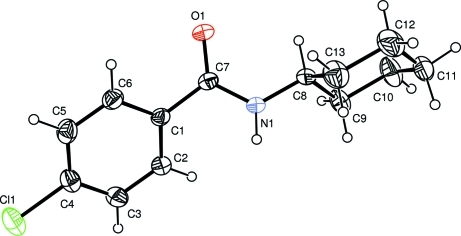
ORTEP-3 (Farrugia, 1997) drawing of (I) with displacement ellipsoids plotted at 30% probability level.

## supplementary crystallographic information

### Comment

*N*-Substituted benzamides, *e.g.*, declopramideare, are well known anticancer compounds and the mechanism of benzamide-induced apoptosis has been studied, (Olsson *et al.*, 2002). N-substituted benzamides inhibit the activity of nuclear factor-B and nuclear factor of activated T cells (Lindgren *et al.*, 2001). Various N-substituted benzamides exhibit potent antiemetic activity (Vega-Noverola *et al.*, 1989), while heterocyclic benzanilide are potassium channel activators (Calderone *et al.*, 2006). *N*-Alkylated 2-nitrobenzamides are intermediates in the synthesis of dibenzo[b,e][1,4]diazepines (Zhichkin *et al.*, 2007) and *N*-Acyl-2-nitrobenzamides are precursors of 2,3-disubstitued 3*H*-quinazoline-4-ones (Beccalli *et al.*, 2005). As part of our work on the structure of benzanilides and related compounds, in this paper, we report the crystal structure of the title compound, (I).

The molecular structure of (I) is presented in Fig. 1. The molecular dimensions in (I) are normal (CSD version 5.30; Allen, 2002). The six-membered ring adopts a chair conformation with puckering parameters: *Q* = 0.576 (3) Å, θ = 0.1 (3)° and φ = 8(15)° (Cremer & Pople, 1975). The structure is stabilized by hydrogen bonding (N1–H1···O1) forming chains of molecules along the *b*-axis (details are in Table 1). The crystal structures of closely related compounds have been reported (Saeed *et al.*, 2008; Jones & Kuś, 2004).

### Experimental

4-Chlorobenzoyl chloride (5.4 mmol) in CHCl_3_ was treated with cyclohexylamine (21.6 mmol) under a nitrogen atmosphere at reflux for 3 h. Upon cooling, the reaction mixture was diluted with CHCl_3_ and washed consecutively with aq 1 *M* HCl and saturated aq NaHCO_3_. The organic layer was dried over anhydrous magnesium sulfate and concentrated under reduced pressure. Crystallization of the residue in CHCl_3_ afforded the title compound (87%) as colorless needles: Anal. calcd. for C_13_H_16_ClNO,: C, 65.68; H, 6.78; N, 5.89%; found: C, 65.61; H, 6.80; N, 5.91%.

### Refinement

All the H-atoms were visible in the difference Fourier maps, they were included in the refinements at geometrically idealized positions with N—H = 0.88 Å and C—H distances = 0.95 - 0.99 Å, and *U*_iso_ = 1.2 times *U*_eq_ of the atoms to which they were bonded. The final difference map was free of chemically significant features.

### Crystal data

**Table:**

C_13_H_16_ClNO	*F*(000) = 504
*M**_r_* = 237.72	*D*_x_ = 1.269 Mg m^−^^3^
Monoclinic, *P*2_1_/*c*	Mo *K*α radiation, λ = 0.71073 Å
Hall symbol: -P 2ybc	Cell parameters from 3651 reflections
*a* = 14.755 (14) Å	θ = 3.9–26.0°
*b* = 5.043 (7) Å	µ = 0.29 mm^−^^1^
*c* = 16.818 (16) Å	*T* = 173 K
β = 96.13 (6)°	Needle, colorless
*V* = 1244 (2) Å^3^	0.12 × 0.08 × 0.06 mm
*Z* = 4	

### Data collection

**Table:**

Bruker APEXII CCD diffractometer	2388 independent reflections
Radiation source: fine-focus sealed tube	1497 reflections with *I* > 2σ(*I*)
graphite	*R*_int_ = 0.034
φ and ω scans	θ_max_ = 26.0°, θ_min_ = 3.9°
Absorption correction: multi-scan (*SORTAV*; Blessing, 1997)	*h* = −18→17
*T*_min_ = 0.967, *T*_max_ = 0.983	*k* = −6→4
3651 measured reflections	*l* = −20→20

### Refinement

**Table:**

Refinement on *F*^2^	Primary atom site location: structure-invariant direct methods
Least-squares matrix: full	Secondary atom site location: difference Fourier map
*R*[*F*^2^ > 2σ(*F*^2^)] = 0.051	Hydrogen site location: inferred from neighbouring sites
*wR*(*F*^2^) = 0.151	H-atom parameters constrained
*S* = 1.06	*w* = 1/[σ^2^(*F*_o_^2^) + (0.0665*P*)^2^ + 0.3764*P*] where *P* = (*F*_o_^2^ + 2*F*_c_^2^)/3
2388 reflections	(Δ/σ)_max_ < 0.001
145 parameters	Δρ_max_ = 0.16 e Å^−^^3^
0 restraints	Δρ_min_ = −0.21 e Å^−^^3^

### Special details

**Table:**

Geometry. All e.s.d.'s (except the e.s.d. in the dihedral angle between two l.s. planes) are estimated using the full covariance matrix. The cell e.s.d.'s are taken into account individually in the estimation of e.s.d.'s in distances, angles and torsion angles; correlations between e.s.d.'s in cell parameters are only used when they are defined by crystal symmetry. An approximate (isotropic) treatment of cell e.s.d.'s is used for estimating e.s.d.'s involving l.s. planes.
Refinement. Refinement of *F*^2^ against ALL reflections. The weighted *R*-factor *wR* and goodness of fit *S* are based on *F*^2^, conventional *R*-factors *R* are based on *F*, with *F* set to zero for negative *F*^2^. The threshold expression of *F*^2^ > σ(*F*^2^) is used only for calculating *R*-factors(gt) *etc*. and is not relevant to the choice of reflections for refinement. *R*-factors based on *F*^2^ are statistically about twice as large as those based on *F*, and *R*- factors based on ALL data will be even larger.

### Fractional atomic coordinates and isotropic or equivalent isotropic displacement parameters (Å^2^)

**Table:**

	*x*	*y*	*z*	*U*_iso_*/*U*_eq_	
Cl1	0.77571 (5)	0.29595 (19)	0.35603 (5)	0.0739 (3)	
O1	0.35835 (12)	0.8003 (3)	0.37556 (11)	0.0489 (5)	
N1	0.32675 (14)	0.3654 (4)	0.38965 (14)	0.0491 (6)	
H1	0.3492	0.2036	0.3922	0.059*	
C1	0.48155 (15)	0.4944 (5)	0.37540 (13)	0.0369 (6)	
C2	0.52269 (17)	0.2853 (5)	0.41805 (15)	0.0462 (6)	
H2	0.4884	0.1813	0.4512	0.055*	
C3	0.61349 (18)	0.2254 (6)	0.41296 (16)	0.0529 (7)	
H3	0.6419	0.0831	0.4431	0.063*	
C4	0.66205 (17)	0.3746 (6)	0.36373 (15)	0.0489 (7)	
C5	0.62262 (18)	0.5866 (6)	0.32108 (16)	0.0539 (7)	
H5	0.6569	0.6895	0.2876	0.065*	
C6	0.53255 (18)	0.6464 (5)	0.32789 (15)	0.0474 (6)	
H6	0.5051	0.7938	0.2996	0.057*	
C7	0.38370 (16)	0.5666 (5)	0.38013 (13)	0.0388 (6)	
C8	0.22970 (16)	0.3992 (5)	0.39603 (16)	0.0484 (7)	
H8	0.2181	0.5912	0.4058	0.058*	
C9	0.20142 (17)	0.2430 (7)	0.46572 (16)	0.0564 (8)	
H9A	0.2371	0.3025	0.5157	0.068*	
H9B	0.2142	0.0524	0.4582	0.068*	
C10	0.0997 (2)	0.2819 (9)	0.4724 (2)	0.0789 (11)	
H10A	0.0816	0.1731	0.5171	0.095*	
H10B	0.0881	0.4702	0.4845	0.095*	
C11	0.04290 (19)	0.2051 (7)	0.3968 (2)	0.0735 (10)	
H11A	−0.0221	0.2421	0.4022	0.088*	
H11B	0.0494	0.0126	0.3875	0.088*	
C12	0.0718 (2)	0.3567 (8)	0.3267 (2)	0.0819 (11)	
H12A	0.0588	0.5476	0.3333	0.098*	
H12B	0.0360	0.2949	0.2770	0.098*	
C13	0.1742 (2)	0.3193 (7)	0.31917 (18)	0.0699 (9)	
H13A	0.1864	0.1313	0.3072	0.084*	
H13B	0.1920	0.4289	0.2746	0.084*	

### Atomic displacement parameters (Å^2^)

**Table:**

	*U*^11^	*U*^22^	*U*^33^	*U*^12^	*U*^13^	*U*^23^
Cl1	0.0443 (4)	0.0948 (7)	0.0840 (6)	0.0035 (4)	0.0128 (4)	−0.0220 (5)
O1	0.0535 (11)	0.0273 (9)	0.0662 (12)	0.0040 (8)	0.0080 (9)	0.0016 (8)
N1	0.0406 (11)	0.0278 (12)	0.0806 (15)	0.0040 (9)	0.0135 (11)	0.0015 (10)
C1	0.0413 (12)	0.0304 (12)	0.0393 (12)	−0.0013 (10)	0.0055 (10)	−0.0056 (10)
C2	0.0445 (13)	0.0408 (14)	0.0535 (15)	0.0020 (12)	0.0069 (11)	0.0053 (12)
C3	0.0475 (15)	0.0517 (17)	0.0589 (16)	0.0078 (13)	0.0025 (12)	0.0025 (14)
C4	0.0410 (13)	0.0589 (18)	0.0472 (14)	−0.0017 (13)	0.0063 (11)	−0.0165 (13)
C5	0.0550 (16)	0.0583 (18)	0.0510 (15)	−0.0079 (14)	0.0183 (13)	−0.0018 (14)
C6	0.0527 (15)	0.0415 (16)	0.0486 (14)	−0.0019 (12)	0.0086 (12)	0.0051 (12)
C7	0.0462 (14)	0.0305 (14)	0.0399 (13)	0.0006 (11)	0.0058 (10)	−0.0009 (10)
C8	0.0396 (13)	0.0292 (13)	0.0768 (18)	0.0047 (11)	0.0074 (13)	−0.0048 (13)
C9	0.0407 (14)	0.078 (2)	0.0518 (15)	0.0046 (14)	0.0100 (12)	−0.0119 (14)
C10	0.0473 (16)	0.113 (3)	0.080 (2)	0.0028 (18)	0.0212 (15)	−0.016 (2)
C11	0.0400 (15)	0.067 (2)	0.113 (3)	−0.0003 (14)	0.0070 (17)	−0.018 (2)
C12	0.0597 (19)	0.088 (3)	0.091 (2)	0.0080 (18)	−0.0240 (17)	−0.005 (2)
C13	0.0609 (18)	0.088 (2)	0.0589 (17)	0.0027 (17)	−0.0039 (14)	0.0122 (17)

### Geometric parameters (Å, °)

**Table:**

Cl1—C4	1.742 (3)	C8—C13	1.510 (4)
O1—C7	1.236 (3)	C8—H8	1.0000
N1—C7	1.338 (3)	C9—C10	1.530 (4)
N1—C8	1.457 (3)	C9—H9A	0.9900
N1—H1	0.8800	C9—H9B	0.9900
C1—C2	1.379 (4)	C10—C11	1.496 (5)
C1—C6	1.386 (3)	C10—H10A	0.9900
C1—C7	1.499 (3)	C10—H10B	0.9900
C2—C3	1.385 (4)	C11—C12	1.505 (5)
C2—H2	0.9500	C11—H11A	0.9900
C3—C4	1.375 (4)	C11—H11B	0.9900
C3—H3	0.9500	C12—C13	1.541 (4)
C4—C5	1.381 (4)	C12—H12A	0.9900
C5—C6	1.379 (4)	C12—H12B	0.9900
C5—H5	0.9500	C13—H13A	0.9900
C6—H6	0.9500	C13—H13B	0.9900
C8—C9	1.508 (4)		
			
C7—N1—C8	123.7 (2)	C8—C9—C10	110.2 (2)
C7—N1—H1	118.1	C8—C9—H9A	109.6
C8—N1—H1	118.1	C10—C9—H9A	109.6
C2—C1—C6	119.1 (2)	C8—C9—H9B	109.6
C2—C1—C7	122.1 (2)	C10—C9—H9B	109.6
C6—C1—C7	118.8 (2)	H9A—C9—H9B	108.1
C1—C2—C3	120.6 (2)	C11—C10—C9	111.7 (3)
C1—C2—H2	119.7	C11—C10—H10A	109.3
C3—C2—H2	119.7	C9—C10—H10A	109.3
C4—C3—C2	119.2 (3)	C11—C10—H10B	109.3
C4—C3—H3	120.4	C9—C10—H10B	109.3
C2—C3—H3	120.4	H10A—C10—H10B	107.9
C3—C4—C5	121.2 (3)	C10—C11—C12	110.8 (3)
C3—C4—Cl1	119.2 (2)	C10—C11—H11A	109.5
C5—C4—Cl1	119.6 (2)	C12—C11—H11A	109.5
C6—C5—C4	118.8 (2)	C10—C11—H11B	109.5
C6—C5—H5	120.6	C12—C11—H11B	109.5
C4—C5—H5	120.6	H11A—C11—H11B	108.1
C5—C6—C1	121.0 (3)	C11—C12—C13	111.3 (3)
C5—C6—H6	119.5	C11—C12—H12A	109.4
C1—C6—H6	119.5	C13—C12—H12A	109.4
O1—C7—N1	122.7 (2)	C11—C12—H12B	109.4
O1—C7—C1	121.0 (2)	C13—C12—H12B	109.4
N1—C7—C1	116.3 (2)	H12A—C12—H12B	108.0
N1—C8—C9	110.6 (2)	C8—C13—C12	110.1 (3)
N1—C8—C13	110.7 (2)	C8—C13—H13A	109.6
C9—C8—C13	110.9 (2)	C12—C13—H13A	109.6
N1—C8—H8	108.2	C8—C13—H13B	109.6
C9—C8—H8	108.2	C12—C13—H13B	109.6
C13—C8—H8	108.2	H13A—C13—H13B	108.1
			
C6—C1—C2—C3	−0.7 (4)	C6—C1—C7—O1	−32.8 (3)
C7—C1—C2—C3	−179.3 (2)	C2—C1—C7—N1	−34.0 (3)
C1—C2—C3—C4	−1.0 (4)	C6—C1—C7—N1	147.4 (2)
C2—C3—C4—C5	1.7 (4)	C7—N1—C8—C9	−132.2 (3)
C2—C3—C4—Cl1	−178.9 (2)	C7—N1—C8—C13	104.5 (3)
C3—C4—C5—C6	−0.6 (4)	N1—C8—C9—C10	179.5 (2)
Cl1—C4—C5—C6	−180.0 (2)	C13—C8—C9—C10	−57.3 (3)
C4—C5—C6—C1	−1.2 (4)	C8—C9—C10—C11	56.9 (4)
C2—C1—C6—C5	1.8 (4)	C9—C10—C11—C12	−56.0 (4)
C7—C1—C6—C5	−179.5 (2)	C10—C11—C12—C13	55.5 (4)
C8—N1—C7—O1	−0.3 (4)	N1—C8—C13—C12	−179.8 (3)
C8—N1—C7—C1	179.5 (2)	C9—C8—C13—C12	57.0 (3)
C2—C1—C7—O1	145.9 (2)	C11—C12—C13—C8	−56.1 (4)

### Hydrogen-bond geometry (Å, °)

**Table:**

*D*—H···*A*	*D*—H	H···*A*	*D*···*A*	*D*—H···*A*
N1—H1···O1^i^	0.88	2.06	2.901 (5)	160

Symmetry codes: (i) *x*, *y*−1, *z*.
